# Urine Bag as a Modern Day Matula

**DOI:** 10.5402/2013/215690

**Published:** 2013-05-23

**Authors:** Stalin Viswanathan

**Affiliations:** Department of General Medicine, Indira Gandhi Medical College, Kathirkamam, Pondicherry 605009, India

## Abstract

Since time immemorial uroscopic analysis has been a staple of diagnostic medicine. It received prominence during the middle ages with the introduction of the matula. Urinary discoloration is generally due to changes in urochrome concentration associated with the presence of other endogenous or exogenous pigments. Observation of urine colors has received less attention due to the advances made in urinalysis. A gamut of urine colors can be seen in urine bags of hospitalized patients that may give clue to presence of infections, medications, poisons, and hemolysis. Although worrisome to the patient, urine discoloration is mostly benign and resolves with removal of the offending agent. Twelve urine bags with discolored urine (and their predisposing causes) have been shown as examples. Urine colors (blue-green, yellow, orange, pink, red, brown, black, white, and purple) and their etiologies have been reviewed following a literature search in these databases: Pubmed, EBSCO, Science Direct, Proquest, Google Scholar, Springer, and Ovid.

## 1. Introduction

For thousands of years physicians had diagnosed their patients' illnesses by examining a urine sample [1]. Uroscopy predated even Hippocrates and began during the Babylonian and Egyptian civilizations [2]. About 20 colors had been described even as early as 100 B.C. in some Sanskrit medical texts. *De Urinis* by Theophilus Protospatharius in the 7th century was hugely influential in uroscopic diagnosis after having demonstrated a range of urinary colors [2]. In the 12th century, Gilles de Corbeil introduced the *matula* to assess color, consistency, and the clarity of urine. The urine was usually examined under direct sunlight [2]. Occasionally the urine was tasted. The matula became a part of the doctor's armamentarium and since then, urine examination had been widely used in the diagnosis of diseases [3]. Physicians began carrying a urinal on horsebacks as a professional trademark [1]. Uroscopy achieved a prominent position in diagnostic medicine during the middle ages after the publication of *Fasciculus Medicinae* by Johannes de Ketham [4]. His illustrated medical text showed a large urine wheel with various urinary shades (~21) that could be matched to a diagnosis [4].

Urochrome gives the normal yellow color to urine [5]. To a lesser extent, urobilin and uroerythrin also contribute towards normal urinary color [6]. Changes in urinary concentration, pH, and metabolic parameters cause color variations in urine [7]. Thudicom characterized the pigment urochrome in the 19th century. Changes in urine color depend on either the increase or decrease in urochrome or the presence of other pigments [8]. Occasionally urine needs exposure to air to develop discoloration as in the case of alkaptonuria. Pigments are either exogenous or endogenous in origin [6]. Endogenous pigments include hemoglobin, myoglobin, bilirubin, uric acid, and homogentisic acid. Exogenous causes are mostly related to medications (e.g., propofol), dyes (e.g., indigo), food (e.g., beet), and poisons (e.g., phenol).

Urine color was thought to reflect the imbalance in the various humors [4]. Depending on the urine color, descriptions of four temperaments (choleric, melancholic, sanguineous, and phlegmatic) were given in the de Katham wheel [4]. Though discoloration is mostly benign and evokes curiosity among physicians, it is disconcerting to patients. Urinary discoloration (e.g., black) was considered lethal in the times of Hippocrates but it may have been to hemolytic crises related to Falciparum malaria [9]. Hospitalized patients with urinary catheters may develop discoloration of urine in hospital due to intrinsic pigments (hematuria, myoglobinuria), medications (intravenous and oral), and dyes used for procedures (e.g., methylene blue). Patients presenting to hospital with a urinary infection or poisoning may have discoloration when catheterized. Both these patient groups with a urine-bag would offer a chance to the physician to observe urinary discoloration. Patients on long-term urinary catheters can present with purple urine-bag syndrome but they would generally have been residents of long-term care facilities.

**Figure 1:**
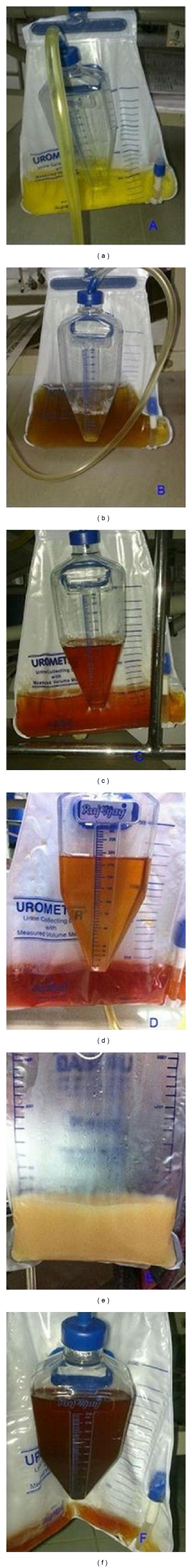
(a) Yellow urine following intravenous vitamin **B** supplements in alcoholic hepatitis. (b) Green urine following organophosphate poisoning. (c) Orange urine—after antituberculous therapy in tuberculous meningitis. (d) Pinkish urine—following hemolysis in cerebral malaria. (e) Pyuria—*Escherichia coli* urosepsis. (f) Cola colored urine—transfusion reaction-related hemolysis.

**Figure 2:**
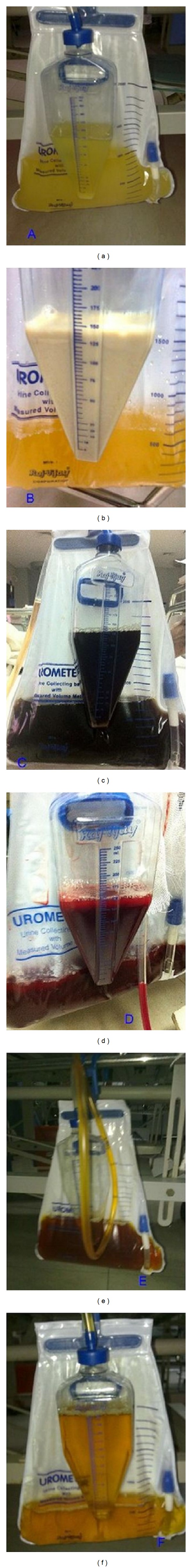
(a) Green urine—*Pseudomonas*-related urinary tract infection. (b) Milky white urine—in Pseudomonas urinary tract infection with chronic kidney disease. (c) Black urine—in Paraphenylenediamine dye intoxication. (d) Red urine—in scrub typhus-related disseminated intravascular coagulation. (e) Orange-brown urine—in a patient with obstructive jaundice and being administered multivitamins. (f) Orange urine—diabetes, urinary tract infection receiving pyridium.

In Figures 1 and 2 there are twelve situations (in the intensive care unit), where urinary discoloration was observed in the author's institution. Discoloration was associated with infections *(Pseudomonas, E. coli*), drugs (vitamins, rifampicin, and pyridium), poisons (organophosphates, paraphenylenediamine), hemolysis (transfusion related, malaria), and jaundice. Barring two instances (*Pseudomonas* infection with chronic kidney disease and scrub typhus-related disseminated intravascular coagulation) all patients survived to discharge from hospital. The various urinary colors reported in the literature are reviewed. The following databases were searched: Pubmed, EBSCO, Science Direct, Proquest, Google Scholar, Springer, and Ovid with search words *urinary discoloration, urine colors, uroscopy, black urine, green urine, *and *urine-bag discoloration.*

## 2. Yellow Urine

Yellow had been associated with a sanguine personality and is due to the presence of bilirubin and urobilinogen [4]. Dehydration, metabolic disorders like diabetes and hypothyroidism, and infections associated with *Serratia marcescens *can cause yellow urine [4]. Urine color has been studied as an indicator of dehydration in ICU patients but has not been found to be reliable [10]. 

## 3. Orange Urine

Orange urine may be the first sign of Lesch Nyhan syndrome in infants. This occurs due to the precipitation of uric acid in urine that gives a pinkish or orangish color to the urine [11]. Increasing pH of the urine reduces precipitation [12]. Vitamin supplements and rifampicin commonly cause orange discoloration.

## 4. Pink Urine

This was associated with a choleric personality [4]. Most causes of red urine including intrinsic pigments and foodstuff also cause pink urine. Uric acid has often been reported in case studies as a reason for pink urine. Uricosuria in obese patients following gastric bypass surgery has led to orangish-pink urine [13]. In these patients, uricosuria is promoted by the effect of increased antidiuretic hormone and surgical-stress-related corticosteroids which leads to precipitation of uric acid crystals [14]. Higher rates of purine synthesis and subsequent uric acid synthesis can lead to pink urine in obese patients (without surgical stress) according to one study [14]. Lower urine volumes, lower pH of urine, and higher osmolality may lead to precipitation [14]. Propofol can indirectly lead to increased urate excretion and produce pink urine instead of green urine which is being more commonly reported. 

## 5. Red Urine

This was linked with having a choleric temperament [4]. Hematuria, hemoglobinuria, and myoglobinuria are the common causes of hematuria. Urine color has been suggested to evaluate the intensity of Schistosoma haematobium infection in communities where the facilities for parasitological diagnosis are difficult [32]. Red urine can give a clue to an aortic-atrial fistula [33] and percussion hemoglobinuria (in people playing percussion instruments with bare hands) [34]. The latter is similar to march hemoglobinuria where intravascular hemolysis and saturation of haptoglobin lead to filtration of free hemoglobin in the kidney. A positive urine dipstick sample (for blood) can be sent for urine microscopy to differentiate hemoglobinuria versus hematuria [33]. Beginning with a reticulocyte count and peripheral smear, bilirubin, haptoglobin, lactate dehydrogenase, and plasma free hemoglobin can rule out hemolysis [33]. A negative urine dipstick examination for hemoglobin in a patient with red urine generally suggests either food pigments, porphyria, or medications [33]. About 14% of people develop reddening of their urine after eating beetroots [35]. Betalain, a pigment in beet, is absorbed from the colon and excreted in urine [35]. Increased beeturia occurs when coingested with high oxalate-containing foods like oysters. Oxalate prevents the decolorization of beet by stomach acid [35]. Phenolphthalein glucuronide when acted upon by colonic bacteria releases the active drug and causes red urine and stools; hence propensity for misuse when used as a laxative is easily noted. Redness is due to its acidification [35]. Other dyes causing red discoloration are that of food colorings (Table 1). Hydroxocobalamin transmits a transient redness to urine and skin and has been described in one case of cyanide poisoning [23]. Lower urinary tract diseases mostly cause red or pink urine [36]. *Citrobacter sedlakii* [11] and *Serratia marcescens* [4] related infections have been described to cause red urine.

## 6. Green Urine

Green urine, like red, was also associated with a choleric personality [4]. Green colored urine is mostly due to medications [37]. Phenol-containing compounds lead to green urine (and black) [16]. Propofol (2,6 diisopropylphenol) causes green urine due to the presence of either its metabolites, fat components in the additives, or due to urate deposition [21]. Propofol is metabolized in the kidney, liver, and intestines. The cytochrome P450 metabolic enzymes oxidizes, reduces, and hydrolyses propofol prior to its glucuronidation in the liver [38]. This urinary discoloration can worsen at the time of alcohol consumption due to the augmented activity of glucuronyl transferase and cytochrome P450-related clearance of propofol leading to higher concentration of its metabolites [21]. Quinol derivatives in urine following glucuronidation lead to green urine. Nearly 70% of metabolites of propofol are excreted in urine [38]. During treatment of morbid obesity with an intragastric balloon, methylene blue dye is placed in the balloon to detect deflation and leak following color changes in urine [39]. Propofol used as a sedative in endoscopies may simulate a balloon leak due to the formation of green urine and hence care is necessary [39]. Similar confusion may arise with use of these two substances in another setting [40]. The methylene blue dye has been used for pressure sores to locate sinuses during surgery where propofol is also used for induction of anesthesia [40]. 

Other medications that have been reported to cause green urine are amitryptiline, metoclopramide, intravenous phenargan, and cimetidine. This may be due to impaired clearance of drugs in renal impairment [41]. Overdosage of drugs like flupirtine and zaleplon have been reported to produce green urine. Green urine has also been observed in carbofuran poisoning [42]. Phenolic derivatives of this carbamate compound and methocarbamol, a carbamyl ester, lead to green urine [42]. Other pesticides like imazosulfuron (2-chloro-N-[[(4,6-dimethoxy-2-pyrimidinyl)amino]carbonyl]imidazo[1,2-a]pyridine-3-sulfonamide) and mefenacet (2-(2-benzothiazolyloxy)-N-methyl-N-phenylacetamide)) when consumed as a component of herbicide have produced green urine [7]. A mutation in the biliverdin reductase gene prevents conversion of biliverdin to bilirubin and results in the formation of green jaundice-green discoloration of urine and plasma [43]. Similarly, indican excess in Hartnup disease and blue diaper syndrome cause production of indoles that causes green discoloration. Urinary infection with *Pseudomonas aeruginosa* colors urine green (e.g., Figure 2(a)) due to release of pigments like pyocyanin and pyoverdin [17]. Occasionally green urine is seen without infection but with risk factors like neurogenic bladder that require intermittent catheterization [44]. 

## 7. Blue Urine

This has been described in patients with a melancholic personality [4]. Very few causes of blue urine have been described, although any cause of green urine can potentially cause a bluish discoloration. Methylene blue, familial indicanuria, amitryptiline (also green urine), and jering (an Indonesian legume) have been known to cause blue urine. Methylene blue is excreted as leucomethylene blue by the kidneys and is bluish in color [37].

## 8. Brown Urine

 This has been ascribed to a mixture of many corrupted humors [4]. Upper urinary tract involvement leads to tea-colored or black/brown urine [6]. This may be due to presence of hemoglobin or myoglobin. Etiologically, inflammatory diseases like glomerulonephritis, infections (malaria), transfusion reactions (e.g., Figure 1(f)), drugs (primaquine), and toxins (snake venom) causing hemolysis and/or rhabdomyolysis are known to produce brown urine. p-Aminophenol in acetaminophen overdose produces brown urine, due to oxidation of p-aminophenol [25]. Brown urine in phenacetin overdose and in methyldopa usage (due to a metabolite methyldopamine) has also been reported [8, 25]. 

## 9. Black Urine

Black discoloration is generally due to presence of intrinsic pigments in urine like hemoglobin, melanin, porphyrin, or myoglobin. Poisons like cresol and endosulfan can also produce black urine by causing hemolysis and rhabdomyolysis and/or excretion of phenolic metabolites [27, 45]. Paraphenylene diamine (in hair dye) poisoning is probably the commonest cause of black urine (e.g., Figure 2(c)) related to poisoning, with many studies having been reported from Northern Africa and India [46]. Fifteen percent of cases of malignant melanoma have melanuria [47]. There are multiple mechanisms that produce black urine in malignant melanoma. During production of melanin from tyrosine, an intermediate metabolite, 5 : 6 dihydroxy-indole, and other phenolic metabolites of melanogens contribute to the black color [47, 48]. It can also occur due to melanin precursors like dopamine undergoing autooxidation on contact with air to form melanin [49]. Pigments like eumelanin which is brown-black and pheomelanin which is yellow-red are seen in blood of patients with malignant melanoma [17]. Brown-black pigment (eumelanin) or yellow-red pigment (pheomelanin) of melanin can lead to black urine in melanoma [50]. 5-S-Cysteinyldopa, a precursor to pheomelanin detectable early in blood and urine, may give a clue to metastasis [50]. Melanuria is not always associated with diffuse melanosis or metastasis to the urogenital tract since dehydration and prerenal azotemia can also contribute to melanuria [50]. Occasionally, exfoliation of melanoma cells or melanogenuria can contribute to black urine [28]. Urinary excretion of homogentisic acid in patients with alkaptonuria leads to blackening of urine on exposure to oxygen and alkali. Other signs like pigmentation of skin and joints are also observed [47]. Insoluble iron complexes like iron sulphide (FeS) causing black urine following injections of iron-sorbitol-citrate have been reported. Bacterial infection in such patients promote formation of hydrogen sulphide that contributes to the sulphide component of FeS [51]. Urease-producing organisms alkalinize urine and can cause oxidation of certain drugs like levodopa to produce dopaquinone and later melanin [26]. 

## 10. White Urine

This is usually due to proteinuria, urinary infection (e.g., Figures 1(e) and 2(b)), or chyluria. A lymphaticourinary fistula occurs in filariasis and leads to passage of milky urine [52]. 

## 11. Purple Urine Bag Syndrome

Purple urine bag syndrome (PUBS) is not discoloration of urine but rather the color of the urine bag, which was first reported in 1978 [30]. The incidence of purple bag syndrome is about 9.8% in institutionalized patients [53]. PUBS has been associated with infections like *E. coli, Klebsiella, Morganella, Citrobacter, Proteus mirabilis,* and* Providencia (rettgeri and stuartii)*. Phosphatase and sulfatase activity of certain intestinal bacteria convert indoxyl sulfate secreted by the liver to indigo (blue) and indirubin (red) in the urine and a combination of these pigments precipitates in alkaline urine and reacts with the synthetic urine bag to form a purple color [53]. Constipation/bowel stasis, long-term urinary catheterization, and high dietary levels of tryptophan can lead to purple urinary bags. Dehydration, hypovolemia, elderliness and female sex are other risk factors for PUBS [31]. Tryptophan transit is prolonged in chronic constipation and leads to production of indoxyl sulfate in the intestine and its enhanced absorption [54]. Carcinomatosis can also cause PUBS but PUBS does not always lead to mortality and is mostly benign [55]. PUBS usually occurs in alkaline urine but can occur in acidic pH as well. Indicanuria (associated with high tryptophan levels) is not always necessary. 

## 12. Conclusion

Urine-bags in hospitalized patients can reveal a spectrum of colors that may be intriguing to the physician. Due to its location, urine-bags are mostly out of vision of treating clinicians. Observing urine bags may give a clue to underlying illnesses like urinary infections, rhabdomyolysis, renal failure, and side effects (or overdosage) of medications and ingested poisons. Rarely, from a diagnostic viewpoint, discoloration may suggest a malignancy, a urinary sinus and can warn of an intragastric balloon leak. Urinary discoloration in such hospitalized patients is mostly benign and resolves with removal of offending agents and appropriate supportive therapy, including antibiotics. Although another de Katham's uroscopic wheel may not be possible at this present age, it would benefit an alert nurse or physician to glance at the urine-bag in place of the *matula*.

## Figures and Tables

**Table 1:** Urinary colors and its causes.

Color	Medications	Dyes	Poisoning	Pigments	Infections	Food
Blue-green	Amitripyiline Azuresin Bromoform Chlorophyll containing breath mints Cimetidine Clioquinol Flupirtine [15] Flutamide [16] Guaiacol [17] Indomethacin Iodochlorhydroxyquin Magnesium salicylate Methocarbamol Mitoxantone Phenylbutazone Phenyl salicylate Promethazine iv Propofol Resorcinol Tetrahydronaphthalene Thymol Tolonium Triamterene Zaleplon [18]	Diagnex blue Evan blue Indigo blue Methylene blue Toluidine blue	Carbamate Imazosulfuron Mefenacet Phenol	Biliverdin Hartnup disease Familial indicanuria [4] Meconium aspiration	Pseudomonas	Asparagus food colorings Jering

Yellow	Vitamins			Diabetes Hypothyroidism Bilirubin Urobilinogen	*Serratia marcescens *	Carrots

Orange	Phenazopyridine [19] Phenolphthalein Rifampicin Vitamin B Vitamin C Warfarin			Uricosuria [13]	*Citrobacter sedlakii* [20]	Beet Carrots

Pink	Propofol [21]	Phenolphthalein		Uric acid Haemoglobin Porphyria Myoglobin		Beets Blackberry Rhubarb Red clover Beefsteak juice

Red	Azosulfamide Azuresin Anthraquinone Chlorpromazine Chloroquine [22] Deferoxamine Hydroxocobalamin [23] Ibuprofen Iron sorbitol Metronidazole Nitrofurantoin Phenazopyridine Phenyl salicylate Phenytoin Phenolphthalein Phenothiazines Rifampicin	Evans blue Indigo blue Indigo carmine	Phenol Boric acid Carbolic acid	Hematuria Hemoglobinuria Homogentisic acid Methemoglobin Myoglobinuria Nutcracker syndrome [24] Porphyrin tyrosinosis urates	*Citrobacter sedlakii* [11] *Serratia marcescens* [4]	Beets Blackberries Food coloring (Aniline congo red rhodamine B) Rhubarb Senna

Brown	Acetaminophen [25] Chloroquine Hydroxychloroquine Furazolidone Levodopa Methocarbamol Methyldopa Metronidazole Nitrofurantoin Niridazole Primaquine			Hematuria Hemoglobinuria Myoglobinuria		Senna

Black	Chloroquine Furazolidone Levodopa [26] Metronidazole Primaquine		Endosulfan Phenylenediamine Cresol [27]	Alkaptonuria Hemoglobinuria Malignant melanoma (exfoliation of melanoma cells) Melanuria Melanogenuria [28] Myoglobinuria Porphyrin [29] Rhabdomyolysis Tyrosinuria	*Proteus mirabilis *	Fava beans Rhubarb

White	Propofol			Chyluria Phosphaturia Proteinuria	Pyuria	

Purple					*E. coli Klebsiella Morganella* [30] *Citrobacter* spp. *Proteus mirabilis Providencia rettgeri * *Providencia stuartii * *Alcaligenes* spp. [31]	
